# Analytical Verification Performance of Afirma Genomic Sequencing Classifier in the Diagnosis of Cytologically Indeterminate Thyroid Nodules

**DOI:** 10.3389/fendo.2019.00438

**Published:** 2019-07-04

**Authors:** Yangyang Hao, Yoonha Choi, Joshua E. Babiarz, Richard T. Kloos, Giulia C. Kennedy, Jing Huang, P. Sean Walsh

**Affiliations:** ^1^Research and Development, Veracyte, South San Francisco, CA, United States; ^2^Medical Affairs, Veracyte, South San Francisco, CA, United States; ^3^Department of Clinical Affairs, Veracyte, South San Francisco, CA, United States

**Keywords:** thyroid cancer, RNA-Seq, genomics, analytical verification, molecular diagnostics, lab developed test, clinical robustness, Afirma GSC

## Abstract

**Background:** Fine needle aspiration (FNA) cytology, a diagnostic test central to thyroid nodule management, may yield indeterminate results in up to 30% of cases. The Afirma® Genomic Sequencing Classifier (GSC) was developed and clinically validated to utilize genomic material obtained during the FNA to accurately identify benign nodules among those deemed cytologically indeterminate so that diagnostic surgery can be avoided. A key question for diagnostic tests is their robustness under different perturbations that may occur in the lab. Herein, we describe the analytical performance of the Afirma GSC.

**Results:** We examined the analytical sensitivity of the Afirma GSC to varied input RNA amounts and the limit of detection of malignant signals with heterogenous samples mixed with adjacent normal or benign tissues. We also evaluated the analytical specificity from potential interfering substances such as blood and genomic DNA. Further, the inter-laboratory, intra-run, and inter-run reproducibility of the assay were examined. Analytical sensitivity analysis showed that Afirma GSC calls are tolerant to variation in RNA input amount (5–30 ng), and up to 75% dilution of malignant FNA material. Analytical specificity studies demonstrated Afirma GSC remains accurate in presence of up to 75% blood or 30% genomic DNA. The Afirma GSC results are highly reproducible across different operators, runs, reagent lots, and laboratories.

**Conclusion:** The analytical robustness and reproducibility of the Afirma GSC test support its routine clinical use among thyroid nodules with indeterminant FNA cytology.

## Introduction

The pre-operative testing of thyroid nodules to differentiate benignity from malignancy is primarily based on fine needle aspiration (FNA) biopsy followed by cytological examination of the collected specimen. While several cytological reporting systems exist and share broad similarities, perhaps the most common is The Bethesda System for Reporting Thyroid Cytopathology (1). About 20–30% of sufficient FNA biopsies are considered neither benign nor malignant and collectively are labeled as cytologically indeterminate (2). Given the possibility of thyroid cancer, patients with an indeterminate FNA are often advised to undergo an invasive surgical procedure to remove part, or all, of the thyroid gland. However, about three-quarters of cytologically indeterminate nodules are found to be benign upon surgical pathology evaluation. Several studies have documented the significant impact of unnecessary diagnostic surgery including direct and indirect medical costs, surgical complications, and impaired patient quality of life (3). Thus, we have developed a molecular test that utilizes genomic information from the thyroid nodule, coupled with machine-learned algorithms, to safely avoid unnecessary diagnostic surgery among patients with indeterminate FNA cytology.

Previously, a microarray-based gene expression classifier (GEC) was developed to classify FNAs with indeterminate FNA cytology, for the goal of achieving a high sensitivity and high negative predictive value performance (4, 5). GEC utilized FNA specimens and reduced unnecessary surgeries (6). More recently, the Afirma GSC was developed using next-generation RNA-sequencing data with improved specificity while maintaining both high test sensitivity and negative predictive values (7). The Afirma platform migration provided broader genomic content for assay improvement compared to the GEC: (1) the measurement of RNA expression via RNA sequencing rather than microarray enabled enhanced detection of transcript levels of nuclear/mitochondrial RNAs and changes in genomic copy number, including loss of heterozygosity; (2) the benign vs. malignant (BM) classifier now consists of an ensemble of machine learning classifiers leveraging global expression patterns of a large number of genes and includes Hürthle and Neoplasm cassettes to improve specificity among Hürthle-containing samples (8); (3) In addition to the BM classifier, the Afirma GSC suite includes three other genomic classifiers: a parathyroid (PTA) classifier, a medullary thyroid cancer (MTC) classifier, and a *BRAF* V600E classifier. Finally, the presence of *RET/PTC1* and *RET/PTC3* fusions, which are highly associated with malignancy, are reported when detected. Each of the components is integrated into the GSC diagnostic flow to provide a GSC benign vs. suspicious result (7). These technical improvements result in substantially improved specificity while maintaining >90% sensitivity (7). The improved test specificity of Afirma GSC results in substantially more patients receiving a benign genomic result which facilitates further reductions in unnecessary surgery (9, 10). The improved specificity also increased the GSC positive predictive value which leads to greater confidence in the need for surgery among those with GSC suspicious results (9, 10).

Here we evaluate the analytical validity of the Afirma GSC suite to demonstrate its robustness to various potential technical variables and interferents arising from sample or laboratory processing. It is critical that clinical tests be robust to real-world conditions so that patients receive consistent, accurate results. Because the Afirma GSC was developed as a rule-out test with a high negative predictive value (NPV) and the intent to avoid unnecessary diagnostic surgeries, many of the analytical validity studies were designed to evaluate the potential of conditions that might introduce a false negative result. In this study, we performed analytical sensitivity analyses to evaluate variable total RNA input amounts on classification results, and the impact of mixing benign or adjacent normal samples into malignant samples via limit of detection (LOD) studies. We also performed analytical specificity analyses to evaluate the impact of mixing blood with the FNA specimen, and the impact of mixing genomic DNA content into the FNA specimen due to incomplete separation of DNA from RNA. We evaluated the reproducibility of Afirma GSC result across different laboratories. Finally, we assessed the reproducibility of Afirma GSC results for replicates within runs, between runs, across multiple reagent lots, and performance by multiple operators on different days. We conclude that the Afirma GSC suite shows robust performance across the tested conditions.

## Materials and Methods

### Specimens

Ethics committee approval—This research was approved by the Copernicus Group Independent Review Board (Cary, North Carolina). A waiver of written informed consent was granted regarding de-identified biological materials from the CLIA laboratory. IRB approval and written Informed consent in accordance with the Declaration of Helsinki was provided by all patients whose samples were previously used for training and validation of the Afirma GSC as previously described (7).

Prospective FNA clinical samples were acquired and shipped under controlled temperature and stored at −80°C until extraction. See Walsh et al. (11) for a description of normal adjacent tissue and blood samples used in this study.

### RNA Extraction, Library Preparation and RNA Sequencing

The RNA library for clinical FNA specimens was purified, prepared, and sequenced as previously described (7). Briefly, total RNA was extracted using the AllPrep Micro Kit (QIAGEN). RNA yield was quantified with the QuantiFluor RNA System (Promega, Madison, WI), and the RNA Integrity Number (RIN) was evaluated using the Bioanalyzer 2100 (Agilent Technology, Santa Clara, CA).

Control samples, including Universal Human Reference (UHR; Agilent, Santa Clara, CA), were included with replicates. Fifteen nanogram of samples and controls were transferred to 96 well-plates and sequencing libraries were prepared with the TruSeq RNA Exome Library Preparation Kit (Illumina, San Diego, CA) automated on the Microlab STAR robotics platform (Hamilton, Reno, NV). Briefly, RNA specimens were fragmented, reversed transcribed, end-repaired, A-tailed, and ligated with adapters, followed by PCR, two rounds of exome capture, and a final PCR amplification according to the manufacturer's recommendation. RNA libraries were then sequenced on NextSeq 500 sequencers (Illumina, San Diego, CA). Sequencing runs with >75% of bases ≥Q30 and <1% phiX error rate were accepted.

### Sequencing Data Processing and Afirma GSC Result Generation

**Figure 1:**
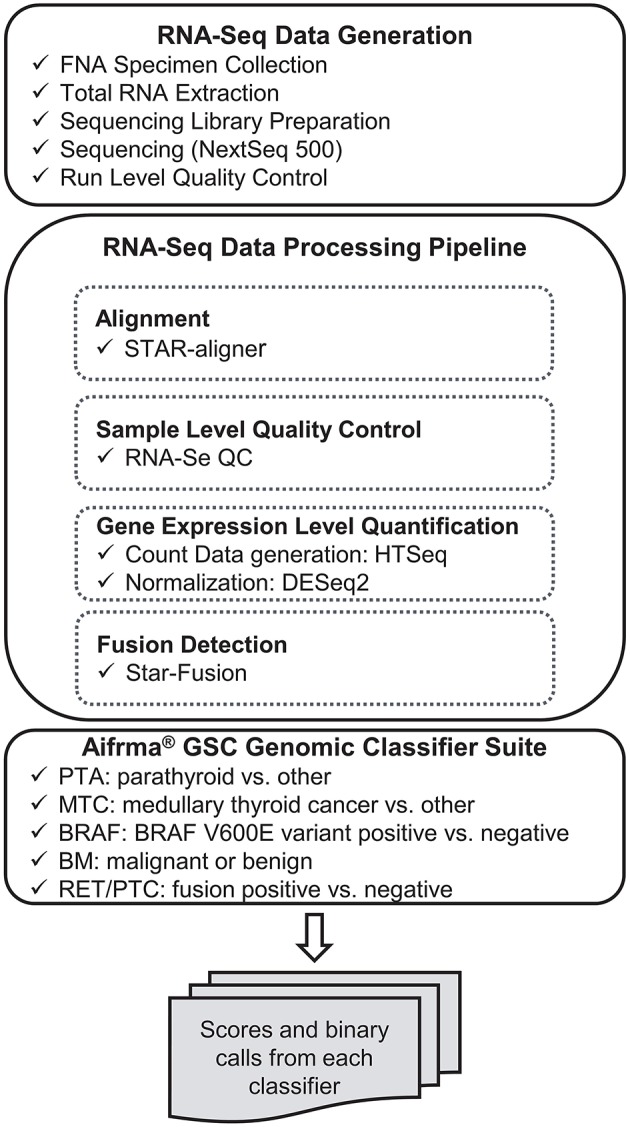
Analytical verification study data generation workflow.

RNA-sequencing data were processed through the bioinformatics pipeline as depicted in the workflow shown in Figure 1. Briefly, raw sequencing data in FASTQ format was mapped to the human reference genome assembly 37 using the STAR RNA-seq aligner (12). Samples passing the quality metrics from RNA-SeQC (13) were analyzed further. Reads data were counted using HTSeq (14). The read count matrix was then normalized using DESeq2 (15) for stabilizing highly variable genes and variation in sequencing depth. Gene fusions were detected using STAR-Fusion (16). The expression level data and fusion-calling results were passed to the Afirma GSC genomic classifier suite and scores and binary calls were generated by each classifier.

### *BRAF* V600E Status

As a reference method, Competitive Allele-Specific Taqman PCR (castPCR™) for *BRAF* c.1799T>A (Thermo Fisher, Waltham, MA) was performed as previously described (7, 17, 18). Samples with a variant allele frequency (VAF) <5% were considered *BRAF* V600 wild type, while VAF ≥5% were considered *BRAF* V600E positive.

### Analytical Verification Study Design

The analytical verification study includes 3 key components: analytical sensitivity, analytical specificity and reproducibility. These components test whether the RNA-sequencing based Afirma GSC genomic classifier suite can maintain the same critical clinical validity and classifier performance of sensitivity ≥90% and NPV ≥90% under conditions of technical variability and interferents potentially encountered in a real-life setting. Afirma GSC BM, *BRAF* V600E, MTC and PTA are all evaluated in a similar fashion.

#### Analytical Sensitivity

- Variability in total RNA input quantity study tests the classifier robustness at five RNA input values: 5, 10, 15, 20, and 30 ng. Both classifier positive and negative samples were sequenced in triplicate for evaluation.
- Dilution of malignant FNA content (limit of detection) tests the dilution effect of malignant (classifier positive) signal by adjacent normal or benign tissue. The dilution levels increase in increments of 20% or 25% for BM, *BRAF* V600E, MTC, and PTA classifiers, and in increments of 5% for RET/PTC fusion detection module.

#### Analytical Specificity

- In a real-life clinical setting, blood is a potential interferent in FNA biopsies. Selected benign, malignant, *BRAF* V600E, MTC and PTA positive samples were mixed with blood proportionally *in vitro* at 0, 25, 50, 75, and 100% to evaluate their impact on the Afirma GSC performance.
- Genomic DNA is another potential interferent and was tested for its possibility to skew the expression level estimations. Both classifier positive and negative samples were mixed with 0% or 30% genomic DNA and sequenced in triplicate for evaluation.

#### Reproducibility

- Inter-laboratory reproducibility tests the concordance of classifier as well as the stability of classifier scores. Samples covering the classifier score range were selected and sequenced in both R&D and CLIA laboratories.
- Assay reproducibility tests the assay stability of classifier calls and scores when samples are run by different operators, on different days and instruments, and across multiple lots of reagents. Selected samples covering each classifier score range were tested in triplicate across three independent experimental runs.

### Analytical Verification Data Analysis

#### Analytical Verification Statistical Analysis

The effects of RNA input amount variation (analytical sensitivity) and genomic DNA interference (analytical specificity) were evaluated using a linear mixed effect model (Equation 1) on the classifier scores (*S*_*ijk*_). Specifically, μ_*i*_ indicates the sample effect and is fitted as a random effect. *b*_*j*_ is the experimental effect and is modeled as a fixed effect. For the input amount variation study, *b*_*j*_ is the input amount (5, 10, 15, 20, or 30 ng); while for the genomic DNA interference study, *b*_*j*_ is the percentage of genomic DNA (0% or 30%). ε_*ijk*_ is the residual, and *k* indicates the technical replicate. Analysis of variance (ANOVA) analysis tests whether the experimental effect is significant (significance level = 0.05).

(1) $$\begin{array}{l} S_{ijk}=\mu_{i}+b_{j}+\varepsilon_{ijk} \end{array}$$

The dilution of malignant FNA (analytical sensitivity) is designed to quantify the impact of interfering classifier negative signals commonly encountered in a real-life clinical setting. To infer the trend of classifier scores at mixture points not tested experimentally (*in vitro*), an *in silico* mixing is performed for each experiment. For each gene *g*, the number of read counts *C*_*gj*_ at mixture percentage *p*_*j*_ (*p*_*j*_ = 0, 0.01, 0.02, …1) is calculated by Equation 2, where *C*_1_ denotes the classifier positive sample and *C*_2_ denotes the classifier negative sample. Then, *C*_*gj*_ is normalized using DESeq2 similarly as other RNA-Seq derived count data.

(2) $$C_{gj}\;=\;p_{j}*C_{1g}\;+\;{\left(1\;-\;p_{j}\right)}^{*}C_{2g}$$

For inter-laboratory reproducibility, the correlation coefficients of samples were computed using Pearson's correlation. The lab effect on classifier scores were estimated by a linear mixed effect model as specified in Equation 1, where *b*_*j*_ indicates the specific laboratory (RD or CLIA). For assay reproducibility, the classifier scores are modeled as:

(3) $$\begin{array}{l} S_{ijk}=\mu_{i}+r_{j}+\mu_{i}:r_{j}+\;\varepsilon_{ijk} \end{array}$$

where μ_*i*_ is the fixed sample effect, and *r*_*j*_ is the run effect, which is modeled as a random effect. μ_*i*_ : *r*_*j*_ is the interaction effect between sample *i* and run *j*, which is modeled as a random effect as well. All 95% confidence intervals for standard deviation (SD) were estimated by bootstrapping.

#### Reproducibility Acceptance Specification Determination

A 3-step simulation study was performed for each classifier to define the tolerable variability level, beyond which, the performance is severely affected.

- (1) Random noise ϵ is generated from the normal distribution ϵ ~ *N*(0, σ^2^), where standard deviation σ spans between 0.36 and 2.10 with an increment of 0.02. Such simulated noise is added to the original classifier scores to create the simulated scores mimicking the classifier being impacted by different levels of noise.
- (2) Performance metrics (for example: sensitivity, specificity, PPA, NPA, etc.) are computed on the simulated scores.
- (3) Steps (1) and (2) are repeated 1,000 times for each noise setting and the median performance metrics are computed.

The maximum score variability tolerated for each classifier is determined with median clinical performance metrics higher or equal to the pre-specified product requirements.

## Results

### Analytical Sensitivity—Total RNA Input Quantity

**Figure 2:**
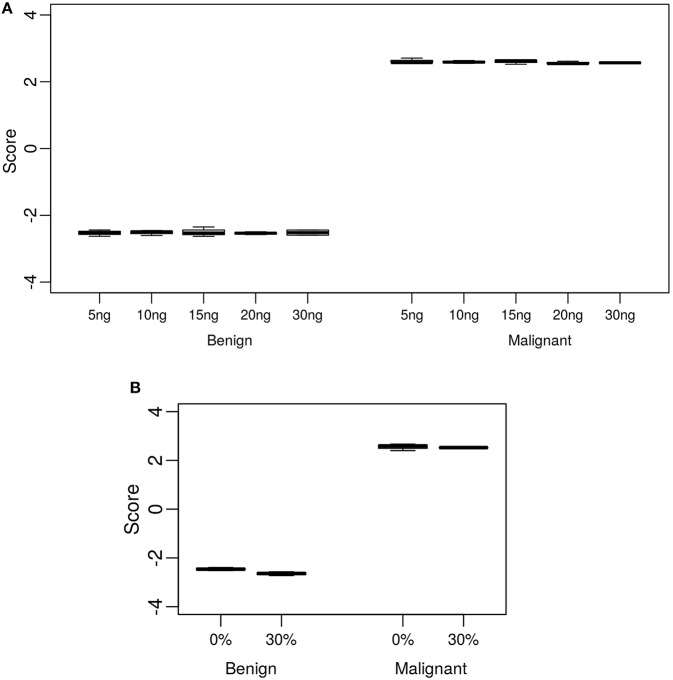
Analytical sensitivity and specificity of the Afirma GSC BM classifier. The y-axis spans the observed score range of the classifier being tested. **(A)** Effect of input mass variation on Afirma GSC scores. Each box represents classifier scores of technical triplicates for either one benign sample or malignant sample. The x-axis shows the total input mass. Overall, GSC scores for each sample did not differ significantly with RNA input amount (*p*-value = 0.97). **(B)** Analytical specificity of the Afirma GSC BM classifier against genomic DNA (gDNA). The x-axis shows the percentage of gDNA spiked into 15 ng of total RNA samples before library preparation. Each box represents classifier scores of all technical replicates for either one benign or malignant sample. Overall, the Afirma GSC BM classifier scores of the same samples with 30% gDNA spike-in are not significantly different from the scores of the corresponding pure RNA samples (*p*-value = 0.064).

The standard input quantity of total RNA to the Afirma® GSC assay is 15 ng. However, the quantity of input material may vary in the lab due to quantitation and pipetting variance. We analyzed the Afirma GSC performance over a range of input amounts wider than expected to occur in the lab to determine the robustness of the test for both classifier positive and negative samples. The titration levels of input mass were evaluated in triplicate at 5, 10, 15, 20, and 30 ng (Figure 2A and Figure S1). There was no significant difference in the GSC BM scores across different input amount when evaluated with a linear mixed effect model (BM classifier: *p*-value = 0.97, Figure 2A). The *BRAF* V600E, MTC, and PTA classifiers were also evaluated (Table S1), and no significant difference in their scores were observed. Finally, a *RET/PTC1* fusion positive sample was tested across the same input amounts and all input amounts tested resulted in *RET/PTC1* true positive calls (Figure S1D). This study showed highly robust analytical sensitivity of the Afirma GSC genomic test to variability in RNA input quantity.

### Analytical Sensitivity—Dilution of Malignant FNA Content (Limit of Detection)

During routine FNA procedures, Adjacent Normal Tissue (ANT) may also be sampled in varying quantities. We sought to define the limit of detection of a classifier positive nodule in the background of adjacent normal tissue or benign tissue. The tolerance of a classifier positive signal to dilution as evaluated using *in vitro* total RNA mixtures from a malignant FNA sample. The limit of detection for classifier positive signals was evaluated at 0, 40, 60, 80, and 100% of adjacent normal tissue or benign tissue as diluted by total RNA mass. The pure adjacent normal tissue was classified as benign by the GSC, whereas all pure malignant FNA samples and different degree of mixtures resulted in malignant GSC calls (Table 1 and Table S2). MTC, PTA and RET-PTC fusions were evaluated in a similar way. Overall, each GSC component could tolerate dilution by more than 75% RNA derived from benign or adjacent normal tissue and still make the correct prediction (Table 1 and Table S2). To understand if the classifier can distinguish the malignant signal in a higher benign background, an *in silico* mixing approach was explored (see Methods). The *in silico* modeling shows that the BM classifier can differentiate a 5% malignant signal in a background of 95% benign RNA (Table 1), suggesting that the presence of malignant signals plays a persistent and dominant role in classification. Additional *in silico* modeling on the PTA and MTC classifiers also revealed robust classification in the background of benign cells, at 15 and 20%, respectively (Table 1). This suggests that when representing a true positive signal, the genomic classifiers are very robust to high level of contamination from true negative content.

**Table 1:** LOD in malignant FNA mixed with benign or adjacent normal tissue.

**Classifier/module**	**RNA mixture**	**LOD in classifier positive FNA**	
		***In vitro***	***In silico***
BM	ANT + Malignant	20%	5%
BM	Benign + Malignant	20%	5%
MTC	Benign + MTC	25%	20%
PTA	Benign + PTA	25%	15%
RET-PTC	Benign + RET-PTC	10%	

*ANT, Adjacent Normal Tissue*.

The limit of detection for the *BRAF* V600E classifier was determined by comparing the classifier call to the *BRAF* V600E variant status derived by castPCR. 264 total samples were evaluated; 62 had a *BRAF* V600E VAF ≥5% and 202 had a *BRAF* V600E 0% ≤ VAF < 5%. The classifier had 100% positive percent agreement (PPA) with *BRAF* V600E castPCR positive samples and a 99% negative percent agreement (NPA) with *BRAF* V600E castPCR negative samples (see Table S3). Therefore, the *BRAF* V600E classifier limit of detection is 5% VAF.

### Analytical Specificity—Blood

Blood may be inadvertently sampled during FNA procedures, with varying amounts of this unintended contaminant observed. To evaluate the tolerance of the GSC classifier to the impact of blood interference, RNA from FNA samples was mixed with RNA derived from a fresh, whole blood sample to create an *in vitro* mixture. The percentages of blood-derived RNA mixtures tested, while holding the total RNA input constant at 15 ng, are 0, 25, 50, 75, and 100%. A fitted sigmoid curve of the GSC BM classifier scores suggested that the malignant sample can be correctly classified with substantially less than the experimentally assessed 25% of the original malignant FNA content (Table 2 and Table S4). The benign sample mixed with blood was correctly classified for all titration points (Table 2 and Table S4). *BRAF* V600E, MTC, and PTA classifiers were evaluated in a similar way. Overall, all classifiers could tolerate presence of >75% blood content in the sample mixture and still make the correct prediction (Table 2 and Table S4). This suggests the genomic classifier is robust to blood contamination.

**Table 2:** Blood interference in Afirma GSC prediction.

**Classifier**	**RNA mixture**	**Maximum interference level of blood**
		***In vitro***
BM	Benign + Blood	100%
BM	Malignant + Blood	75%
*BRAF* V600E	*BRAF* V600E + Blood	75%
MTC	MTC + Blood	75%
PTA	PTA + Blood	75%

### Analytical Specificity—Genomic DNA

Genomic DNA (gDNA) is removed from samples during RNA extraction, however the removal may not be complete in all samples tested. To test the impact of gDNA contamination, we spiked high amounts of gDNA into purified RNA. Testing pure gDNA in the Afirma GSC assay did not produce any sequencing library, and thus the potential for interference in such setting is minimal. To test the impact of gDNA contamination in a sample that produces sequence-able libraries, we evaluated 30% gDNA added to RNA. This level of gDNA contamination can be observed during RIN evaluation in the bioanalyzer traces of RNA (Figure S2A). We reasoned that if such a high level of gDNA contamination were observed in a QC step, but did not interfere with the assay, then lesser amounts of contamination would not affect performance. To experimentally assess the extent of gDNA interference on the Afirma GSC BM results, *in vitro* mixtures were created with one malignant and one benign sample each with 30% gDNA spiked in, while maintaining the total RNA input constant at 15 ng. Afirma GSC scores of the BM classifier for each sample did not differ significantly between samples with and without 30% genomic DNA spiked in (*p*-value = 0.064). As shown in Figure 2B, the observed score differences are very small when shown in the score range of BM classifier. These observations support that the Afirma GSC test is robust against genomic DNA interference. The *BRAF* V600E, MTC classifiers and RET/PTC fusion detection module were evaluated and no significant difference was observed (Table S5 and Figure S2). The PTA classifier had a statistically significant difference (*p* = 0.005), but this observation was due to the extremely reproducible scores and a small shift in the PTA score at 30% gDNA (Table S5 and Figure S2). This score shift represents <1% of the total score space of the PTA classifier and will not have an impact on PTA classification performance.

### Reproducibility—Acceptance Specification

To understand the threshold of variation the Afirma GSC can tolerate, an *in-silico* simulation with increasing levels of random variation was performed. The GSC BM classifier score indicated that the classifier can tolerate a total variation of scores SD ≤ 0.44 from all technical sources without substantially impacting sensitivity and specificity (Table 3). The *BRAF* V600E, MTC, and PTA classifiers were also evaluated in a similar fashion, and the maximum tolerable variation level are summarized in Table 3 column “SD Specification.” As with the GSC BM classifier, these specification values serve as the acceptance thresholds to be compared against the technical variation levels estimated from reproducibility studies.

**Table 3:** Afirma GSC classifier suite reproducibility result summary.

**Classifier**	**Score range**	**Biological variation**		**SD specification**	**Technical variation**					
		**Inter-class**			**Inter-lab**		**Inter-run**		**Intra-run**	
		**N**	**SD (95% CI)**		**N**	**SD (95% CI)**	**N**	**SD (95% CI)**	**N**	**SD (95% CI)**
BM	8	191	1.452 (1.306–1.634)	0.440	80	0.130 (0.114 −0.144)	134	0.274 (0.228–0.310)	134	0.069 (0.053–0.073)
*BRAF* V600E	13	264	3.417 (3.189–3.698)	0.640	42	0.298 (0.249–0.344)	134	0.186 (0.169–0.202)	134	0.133 (0.115–0.153)
MTC	12	211	2.912 (2.424–3.499)	2.000	50	0.174 (0.088–0.253)	53	0.189 (0.114–0.257)	53	0.189 (0.081–0.214)
PTA	10.5	195	1.242 (0.735–1.845)	1.210	50	0.104 (0.090–0.117)	36	0.105 (0.082–0.124)	36	0.078 (0.040–0.079)

“*SD Specification” was derived by in-silico simulation on the training set scores for each classifier. “N” is the sample size for each study. 95% confidence interval is included for all SD estimates*.

### Reproducibility—Inter-laboratory

**Figure 3:**
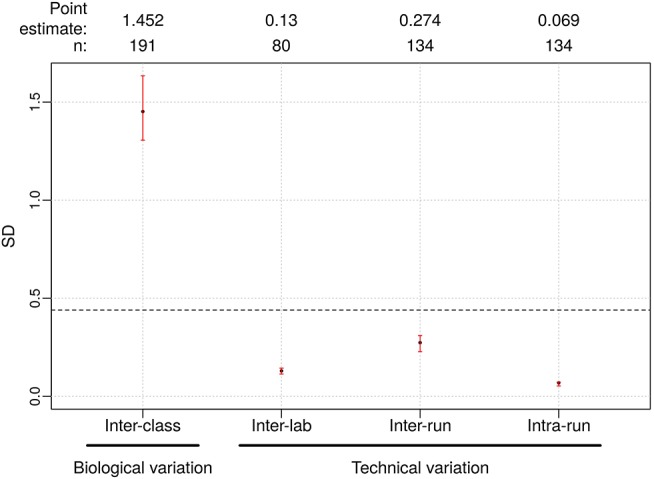
Reproducibility results for Afirma GSC BM classifier. The left most is the biological variation calculated as the inter-class score SD between benign and malignant samples and was computed from all samples passing quality control criteria in the clinical validation study. On the right side, technical variability from different sources were listed. Dashed line: the maximum tolerable level of technical variation in GSC scores derived from simulation (0.44). Black dots: observed values. Vertical red lines: 95% CI. The sample size used to calculate the point estimate and the 95% CI is shown at the top.

The Afirma GSC was developed in the Veracyte R&D laboratory and transferred to the CLIA laboratory for clinical use. To confirm that the two labs achieve the same GSC BM results, a total of 40 unique patients spanning the entire GSC BM score range were used to evaluate the inter-laboratory reproducibility. The Afirma GSC BM classifier call results from the two laboratories were 100% concordant (40 of 40). Additionally, the Afirma GSC BM scores of the 40 samples between the two laboratories were highly correlated (R^2^ = 0.99) and demonstrated strong inter-laboratory reproducibility of the Afirma GSC BM classifier. The inter-laboratory pooled SD of Afirma GSC BM scores was estimated to be 0.130 (95% CI: [0.114, 0.144]), which is substantially below the pre-determined acceptance threshold of SD ≤ 0.44 from *in-silico* simulation (Figure 3). Other classifiers were evaluated in the same fashion and rendered similar results (Table 3 and Figure S3).

### Reproducibility—Assay Inter-run and Intra-run

In a CLIA lab production environment, samples are processed continually by different operators, on different machines, and across different lots of reagents. It is therefore critical to ensure performance stability across these variables to ensure that patients tested over the lifetime of the product receive consistent results. We performed reproducibility studies that examined identical samples run by different operators, on different days and instruments, across multiple lots of reagents.

To evaluate the tolerance of the Afirma GSC BM to experimental variation due to equipment, operator, reagent lot, and days, 15 malignant and benign FNA samples spanning the entire score range were tested in triplicate across three independent experimental runs. The intra- and inter-run reproducibility of the Afirma GSC BM were evaluated using total RNA from 134 assays that passed QC, tested in 3 experimental runs.

The pooled intra-run SD of Afirma GSC BM scores, quantifying variability from technical replication, was estimated to be 0.069 (95% CI: [0.053, 0.073]). The pooled inter-run SD of GSC scores, measuring the total experimental variability other than sample-specific effects, was estimated to be 0.274 (95% CI: [0.228, 0.310]) across all samples in this study. In comparison, the total SD of inter-class scores between benign and malignant samples, reflecting biological difference, was estimated to be 1.452 (95% CI: [1.306, 1.634]) (Figure 3). Observed technical variation of the BM and other classifiers were all below the pre-determined specs from the *in-silico* simulation (Table 3, Figure 3 and Figure S3). RET/PTC fusion positive samples were all called positive in all replicates across all batches. Thus, the Afirma GSC genomic test is highly robust to routinely encountered sequencing operational variations.

## Discussion

The Afirma GSC classifier is a novel genomic diagnostic test that leverages whole transcriptome RNA-sequencing and machine learning methods to accurately predict benign vs. malignant thyroid nodules. It is an enhanced version of its predecessor, the Afirma GEC classifier (5). The clinical validity of the GSC has been established (7). Equally important to clinical validation is the establishment of analytical validation, as outlined by the Evaluation of Genomic Applications in Practice and Prevention (EGAPP) Working Group and the Centers for Disease Control's ACCE (Analytic and Clinical validity, Clinical utility and associated Ethical) Project (19, 20). All analytical validation studies were performed in a prospective manner, whereby the acceptance criteria for each study were determined (1) based on previously approved design requirements and (2) prior to the study being performed in the laboratory. Here we report the analytical validation of the GSC and demonstrate its robustness to various technical variations along the entire pipeline process of plating, library generation, sequencing, and algorithm analysis. More specifically, we show the tolerance of the classifier to the impact of variation in RNA input amount, and heterogenous sample inputs resulting from admixture with blood, genomic DNA, and adjacent normal tissues. Sample collection, storage, and shipping were unchanged from the Afirma GEC and their analytical validity was previously established (11).

Our studies demonstrated that the Afirma GSC classifier is robust to technical variability encountered in routine clinical sample processing. Analytical sensitivity studies showed that classifier calls are not impacted by variation in RNA input deviating from the standard amount (15 ng); it can tolerate variance up to −10 ng to +15 ng (range, 5–30 ng). Clinical sample collection procedures may yield an impure nodule sample due to the needle passing through other tissues on its way to the thyroid nodule of interest. Limit of detection studies here demonstrated that the BM classifier is robust to dilution of malignant FNA content by adjacent normal (80%) and benign FNA content (80%) and *in silico* modeling shows that the BM classifier can tolerate even higher dilution (95%). All other components of the GSC test system also demonstrated significant resistance to the effects of dilution.

Potential interferents such as blood and genomic DNA may also be mixed in the samples due to variability in sample collection, preparation, or biological heterogeneity. Analytical specificity analyses showed that malignant signals remained sufficiently detectable with up to 75% of blood mixed into a malignant sample. Also, no changes in classifier calls were found with up to 30% of genomic DNA in the sample mixture. These results suggest that the Afirma GSC test is robust to heterogenous sample content.

We selected both positive and negative control samples and FNA samples that cover the entire score range to evaluate the reproducibility of GSC test results according to regulatory requirements of genomic applications. We showed that the Afirma GSC scores and calls are reproducible for samples replicated across different reagents, operators, equipment and runs. The accuracy of the test performed at the CLIA-certified laboratory was established by an inter-laboratory comparison study, showing that the test results generated from the CLIA-certified commercial laboratory are consistent with those generated in the R&D laboratory where the test was developed. Combined with the clinical validation study previously published (7), the GSC successfully fulfills the analytic criteria of EGAPP level I.

## Conclusion

The Afirma GSC demonstrates robust reproducibility and analytical performance against technical variability that may arise from clinical sample collection and laboratory processing. These findings support its clinical use among cytologically indeterminant thyroid nodules to inform patient care.

## Data Availability

Restrictions apply to the datasets: The datasets for this manuscript are not publicly available because: the dataset and the research methodologies are proprietary. Requests to access the datasets should be directed to GK, Ph.D.; Giulia@veracyte.com.

## Ethics Statement

This research was approved by the Copernicus Group Independent Review Board (Cary, North Carolina). A waiver of written informed consent was granted regarding de-identified biological materials from the CLIA laboratory. IRB approval and written Informed consent in accordance with the Declaration of Helsinki was provided by all patients whose samples were previously used for training and validation of the Afirma GSC as previously described (7).

## Author Contributions

YH, YC, JB, RK, GK, JH, and PW designed, performed, analyzed, and interpreted the study. YH, JC, JB, RK, GK, JH, and PW implemented the analysis, prepared figures, and drafted the manuscript. YH, JB, RK, GK, JH, and PW edited and revised the manuscript. All authors have read and approved the final manuscript.

### Conflict of Interest Statement

All authors are Veracyte Inc. employees and equity owners.
