# Supplementary material for: Analytical Verification Performance of Afirma Genomic Sequencing Classifier in the Diagnosis of Cytologically Indeterminate Thyroid Nodules
Source: Front Endocrinol (Lausanne). 2019 Jul 4;10:438. doi: 10.3389/fendo.2019.00438 (PMC6620518; doi:10.3389/fendo.2019.00438)
Supplement: Supplementary file 1 [file Data_Sheet_1.PDF]

Supplementary materials

Analytical sensitivity – total RNA input quantity

The classification scores from each Afirma GSC classifier were tested to determine the effect of various RNA input amounts. Different biological samples were used for each classifier (BM classifier: benign and malignant *BRAF*-; *BRAF* V600E classifier: benign and malignant *BRAF*+; MTC classifier: benign and MTC+; PTA classifier: benign and PTA+). A linear mixed effect model (input amount: fixed effect; biological samples: mixed effect) was applied to test the score difference among various RNA input amounts. No significant difference was observed in RNA input amount for BM, *BRAF*, MTC and PTC classifiers (**Table S1 and Figure S1 A-C**). *RET/PTC1* fusion was detected in all replicates across all input amount, indicating that *RET/PTC1* fusion cassette can tolerate RNA input as low as 5 ng. (**Figure S1 D**).

Table S1: Impact of total RNA input quantity on classification scores

Classifier	P-value
BM	0.971
BRAF V600E	0.123
MTC	0.141
PTA	0.366

Figure S1

(A) *BRAF* V600E

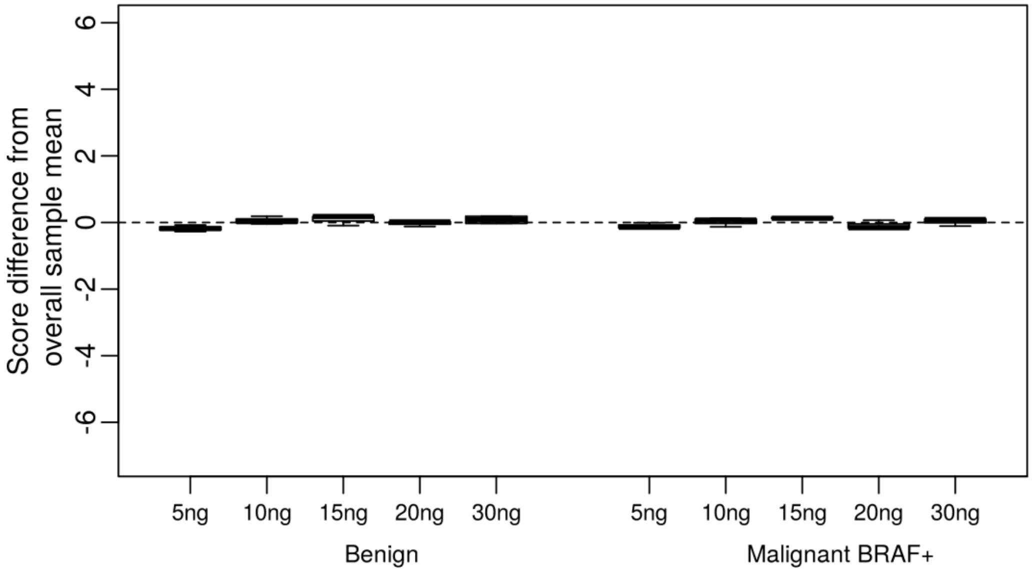

(B) MTC

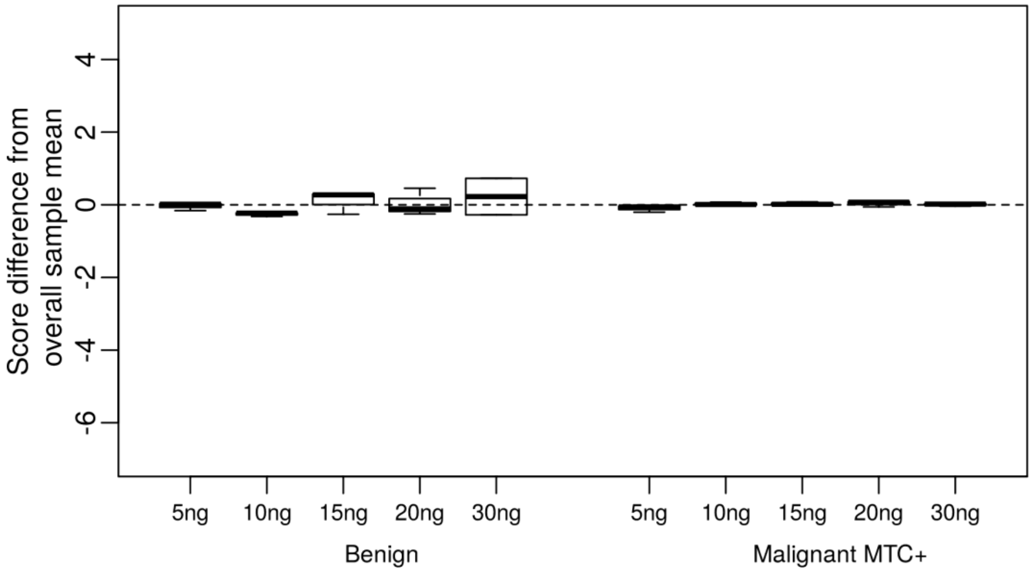

(C) PTA

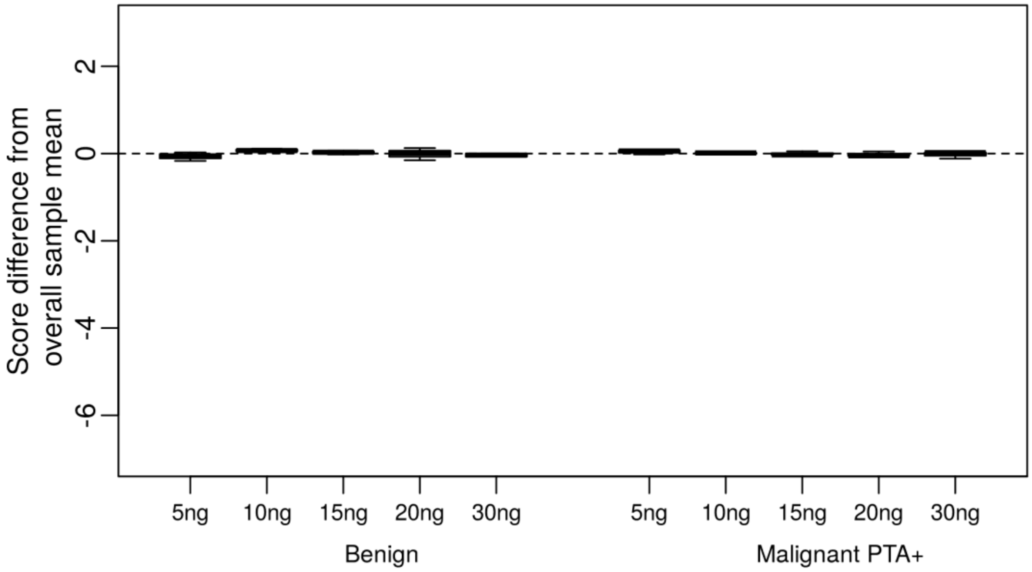

(D) RET/PTC1

	5 ng	10 ng	15 ng	20 ng	30 ng
Rep 1	Positive	Positive	Positive	Positive	Positive
Rep 2	Positive	Positive	Positive	Positive	Positive
Rep 3	Positive	Positive	Positive	Positive	Positive

Figure S1: Impact of total RNA input quantity on classification scores in *BRAF* (A), MTC (B), PTA (C). The X-axis shows the total input mass and the y-axis is on a relative scale, with 0 representing the mean of each sample across all input levels (mean centered), and the y-axis is shown in the observed score range for each classifier. Each box represents test results from technical triplicates of (1 benign and 1 malignant) biological samples. (D) *RET/PTC1* detection calls in triplicate for 5, 10, 15, 20, and 30 ng of input material.

Analytical sensitivity – dilution of malignant FNA content (limits of detection)

MTC, PTA and *RET/PTC1* positive samples were mixed with benign or adjacent normal tissues proportionally *in vitro*. At each proportion, triplicates were sequenced and scored. A fitted line connected the median of the data points at each proportion level (**Table S2**). The LOD was defined as the minimum proportion where the original positive prediction will be converted to

negative due to the interfering benign or adjacent normal tissues (**Table S2**). Overall, the *in vitro* mixing experiment showed that all classifiers/modules can tolerate over 75% RNA derived from benign or adjacent normal tissue while still making correct calls, suggesting the robustness of Afirma GSC.LOD of the *BRAF* V600E classifier was determined according to Variant Allele Frequency (See Table S3).

Table S2: *In vitro* LOD results.

"0/3" means 0 positive call out of 3 replicates. "3/3" means 3 positive calls out of 3 replicates.

Classifier / module	RNA Mixture	Number of positive calls across proportions of classifier positive											
		0%	5%	10%	15%	20%	25%	40%	50%	60%	75%	80%	100%
BM	ANT + Malignant	0/3				3/3		3/3		3/3			3/3
BM	Benign + Malignant	0/3				3/3		3/3		3/3			3/3
MTC	Benign + MTC	0/3					3/3		3/3		3/3		3/3
PTA	Benign + PTA	0/3					3/3		3/3		3/3		3/3
RET-PTC	Benign + RET-PTC		1/3	3/3	3/3	3/3			3/3				3/3

Table S3: Comparison of *BRAF* V600E classifier status to castPCR

	BRAF V600E castPCR ≥5%	*BRAF* V600E castPCR <5%
BRAF V600E classifier positive	62	2
BRAF V600E classifier negative	0	200
Total	62	202

Analytical specificity – blood

The malignant, *BRAF*, MTC and PTA positive samples were mixed with blood proportionally *in vitro* at 0%, 25%, 50%, 75% and 100%. At each proportion, triplicates were sequenced and scored. A fitted line connected the median of the data points at each proportion level (**Table S4**). We quantified the level of blood interference as the largest proportion where the original positive prediction will be converted to negative due to the interfering blood (**Table 2 and Table S4**).

Overall, all classifiers can tolerate over 75% RNA derived from blood while still making correct calls, suggesting the robustness of Afirma GSC to blood contamination.

Table S4: *In vitro* blood interference results.

"0/3" means 0 positive call out of 3 replicates. "3/3" means 3 positive calls out of 3 replicates.

Classifier / module	RNA Mixture	Number of positive calls across proportions of blood				
		0%	25%	50%	75%	100%
BM	Benign + Blood	3/3	3/3	3/3	3/3	3/3
BM	Malignant + Blood	3/3	3/3	3/3	3/3	0/3
BRAF V600E	BRAF V600E + Blood	3/3	3/3	3/3	3/3	0/3
MTC	MTC + Blood	3/3	3/3	3/3	3/3	0/3
PTA	PTA + Blood	3/3	3/3	3/3	3/3	0/3

Analytical specificity – genomic DNA

To experimentally assess the extent of genomic DNA interference on the classifiers' results, in vitro mixtures were created with one malignant and one benign sample each with 30% gDNA spike-in, while maintaining the total RNA input constant at 15ng. 30% gDNA contamination can be observed in bioanalyzer traces of total RNA used to determine RIN. **Figure S2 A** shows benign RNA ± 30% gDNA and malignant RNA ± 30% gDNA. The gDNA is observed as a peak between the 16S and 28S rRNA peaks, and a higher molecular weight peak. The classification scores of BM, *BRAF*V600E, MTC for each sample did not differ significantly between samples with and without 30% genomic DNA spiked in (**Table S5; Figure S2 B, C**). Although the P-value of the PTA classifier is below 0.05, the mean score shift between 0% and 30% gDNA is 0.09, which is less than 1% of the PTA score range **(Figure S2 D)**. The total SD across 0% and 30% gDNA is 0.075, which is far below the pre-determined spec 1.21 from the *in-silico* simulation. RET/PTC1 calls were robust to gDNA input **(Figure S2 E)**. These results demonstrate that the Afirma GSC is robust against genomic DNA interference.

Table S5: score comparison with and without 30% spike-in genomic DNA

Classifier	P-value (0% vs. 30% genomic DNA)

BM	0.064
BRAF V600E	0.260
MTC	0.852
PTA	0.005

Figure S2

(A) Bioanalyzer plots of gDNA contamination at 30%

Benign RNA Sample

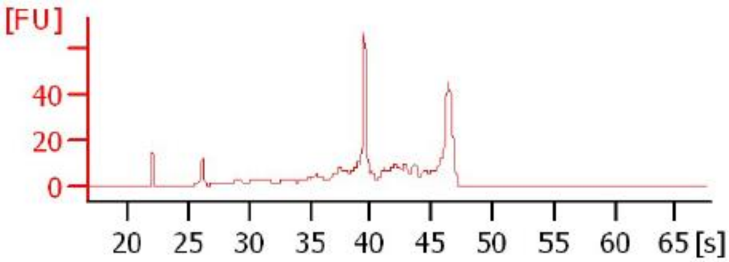

Benign RNA Sample + 30% gDNA

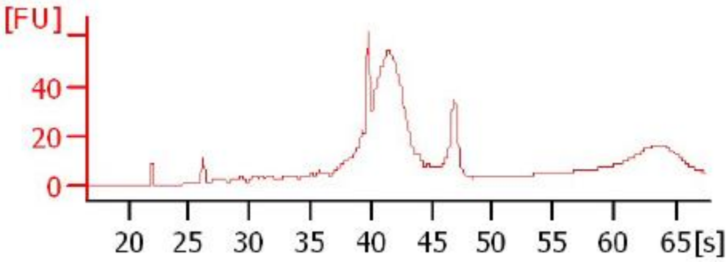

Malignant RNA Sample

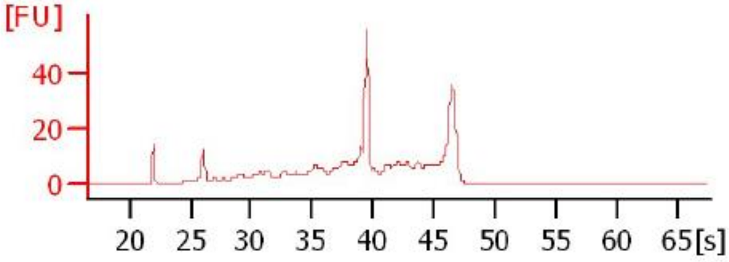

Malignant RNA Sample + 30% gDNA

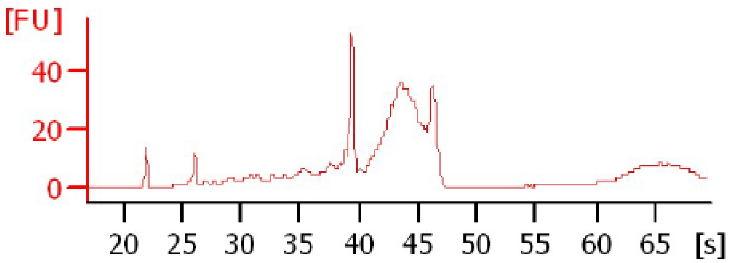

(B) *BRAF V600E*

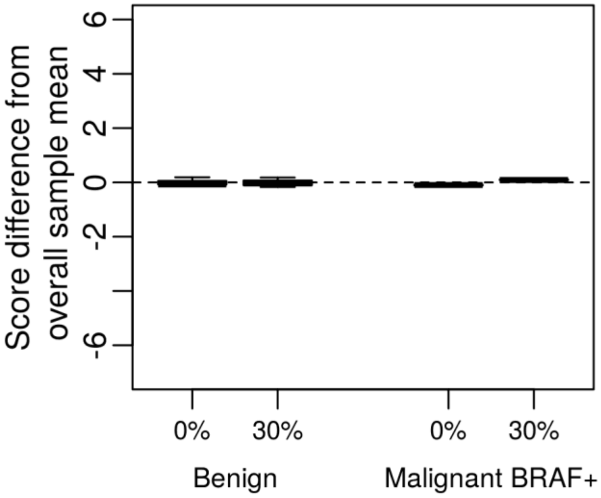

(C) MTC

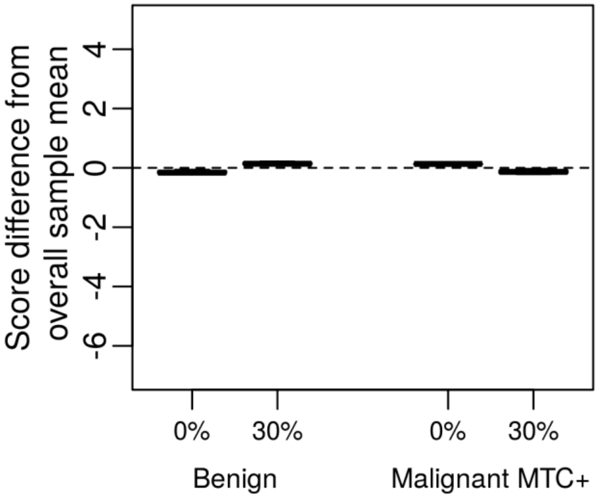

(D) PTA

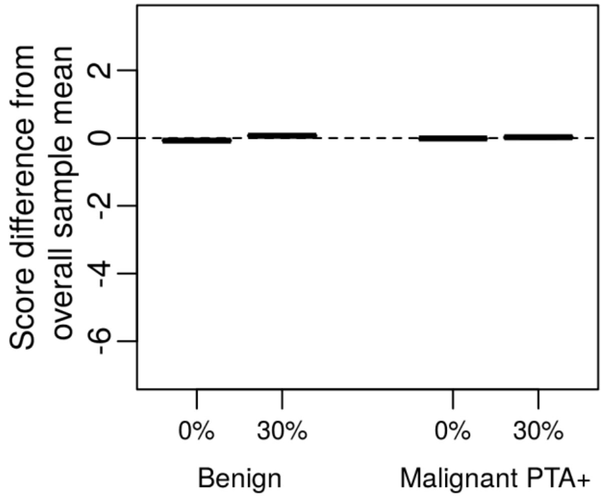

(E) *RET/PTC1* fusion

	RET/PTC1 Negative		*RET/PTC1* Positive	
	0% gDNA	30% gDNA	0% gDNA	30% gDNA
Rep 1	Negative	Negative	Positive	Positive
Rep 2	Negative	Negative	Positive	Positive
Rep 3	Negative	Negative	Positive	Positive

Assay Reproducibility

The technical variation, including inter-lab, inter-run and intra-run SD and the biological variance, inter-class SD, were estimated for the BM, *BRAF*, MTC, and PTA classifiers. Technical variation of the classifiers (inter-lab, inter-run, and intra-run SD) was all below the specifications from the in-silico simulation and substantially lower than the biological variance (inter-class SD) indicating that the assay is robust to technical noise and sensitive to biological signals.

Figure S3

(A) BRAF

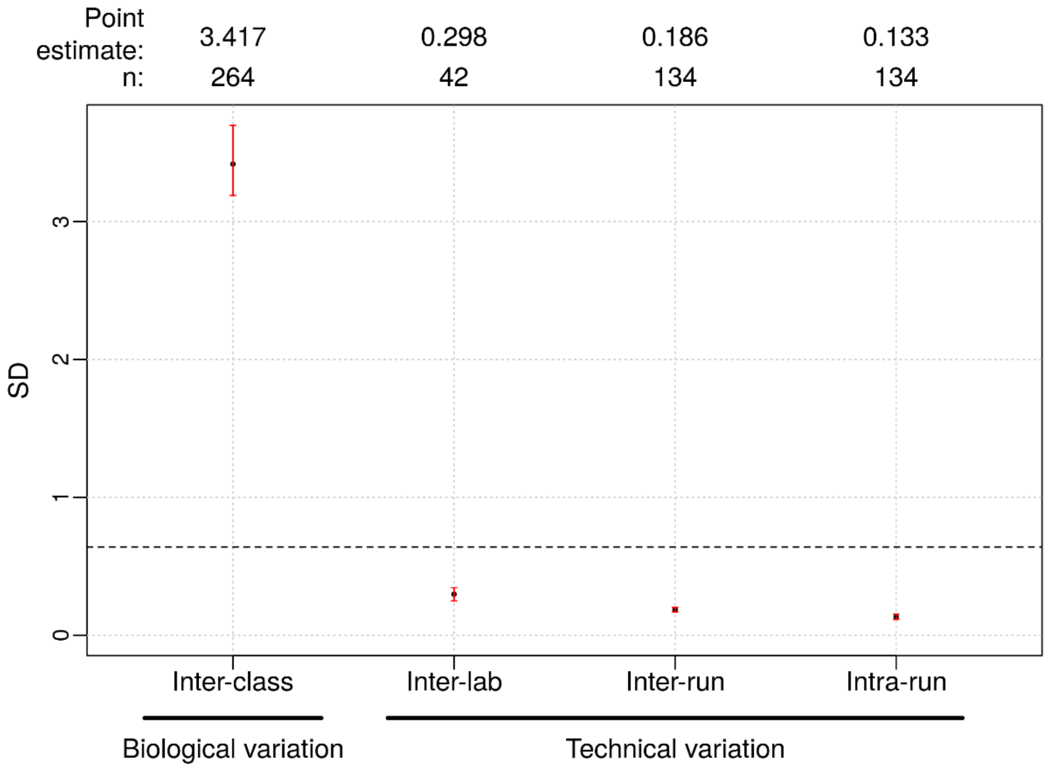
Point estimate:
3.417
0.298
0.186
0.133
n:
264
42
134
134
SD
0
1
2
3
Inter-class
Inter-lab
Inter-run
Intra-run
Biological variation
Technical variation

(B) MTC

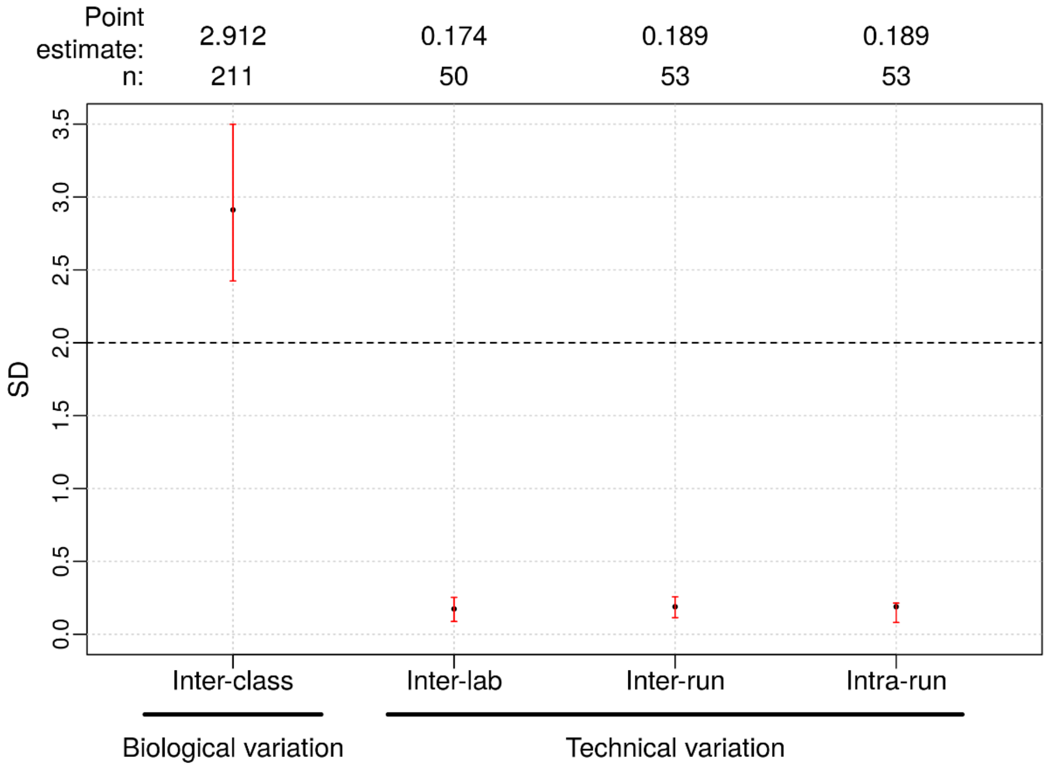
Point estimate:
2.912
0.174
0.189
0.189
n:
211
50
53
53
SD
0.0
0.5
1.0
1.5
2.0
2.5
3.0
3.5
Inter-class
Inter-lab
Inter-run
Intra-run
Biological variation
Technical variation

(C) PTA

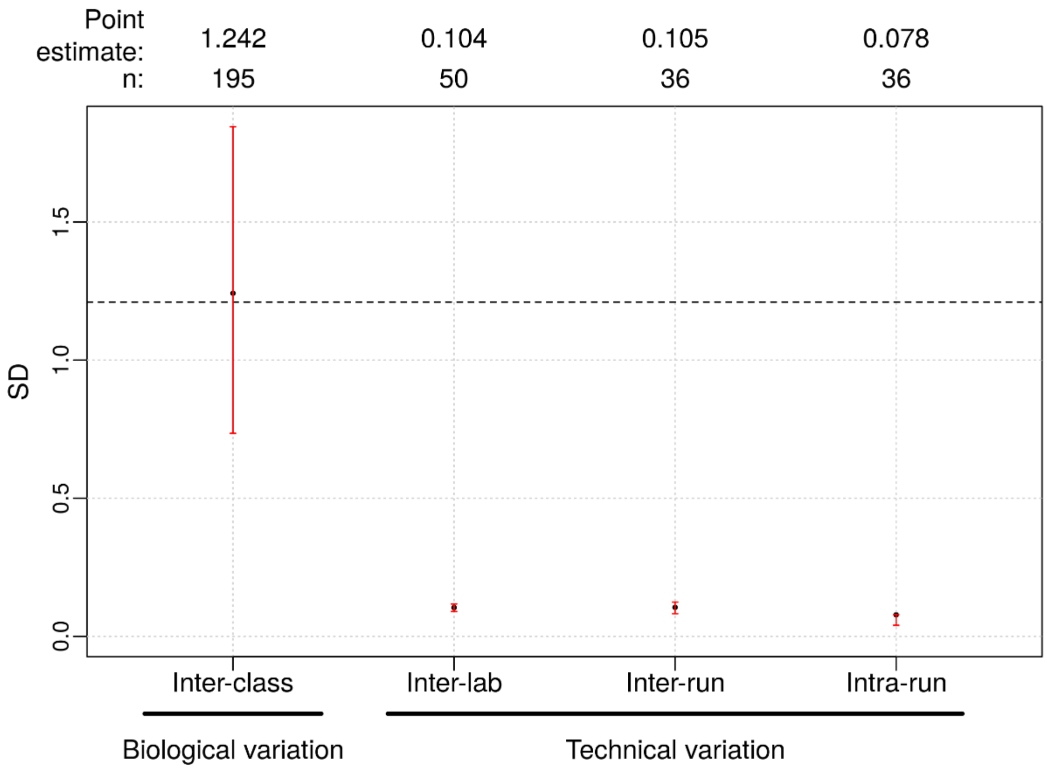

Figure S3 Afirma GSC Score Variability of *BRAF*, MTC and PTA classifiers. The inter-class score SD includes biological variation among different sample types and was computed from all samples passing quality control criteria in the clinical validation study. Dashed line: the maximum tolerable level of variation in GSC scores derived from simulation (Specifications in **Table 3**) Black dots: point estimates. Vertical lines: 95% CI. The number of data points used to calculate each SD (n) is shown at the top.
